# Tourist Experiences at Overcrowded Attractions: A Text Analytics Approach

**DOI:** 10.1007/978-3-030-65785-7_21

**Published:** 2020-11-28

**Authors:** Joanne Yu, Roman Egger

**Affiliations:** 1 Department for Informatics, Technical University of Munich, Garching bei München, Bayern Germany; 2 Smart Tourism Education Platform (STEP) College of Hotel and Tourism Management, Kyung Hee University, Seoul, Korea (Republic of); 3 Department of Tourism and Service Management, MODUL University Vienna, Vienna, Wien Austria; Salzburg University of Applied Sciences, Urstein Süd 1, 5412 Puch Urstein, Salzburg, Austria

**Keywords:** Overtourism, Carrying capacity, User-generated content, Topic model, Sentiment analysis

## Abstract

As a result of travel activities, overtourism has become a global issue. Even after the COVID-19 pandemic, the topic of overtourism would benefit localized overcrowding as a new occurrence in the tourism industry. Since there is no specific measurement to evaluate tourist experiences at crowded attractions, this study aims to explore the perception and feelings of tourists when they visit popular and crowded attractions through topic modeling and sentiment analysis based on TripAdvisor online reviews as of the end of 2019. By investigating the top 10 attractions in Paris, the results present 24 topics frequently discussed by tourists. Examples of some topics related to overtourism are safety, service, queuing, and social interaction. Specifically, tourists felt the most negative towards safety and security among all the identified topics. By bridging overtourism, text analytics, and user-generated-content, this study contributes to the field of tourist experiences and crowd management.

## Introduction

While revenue generated through tourism activities directly contributes to a country’s GDP, negative consequences emerge from the high number of visitors over a period of time. In contemporary tourism, exceeding a destination’s carrying capacity or crowding of tourist sites through day visitors are considered as the precursor of overtourism [1, 2]. Overtourism is harmful to the environment [3], society, and the economy of a destination [4]. One of the indicators of this phenomenon is the perception of crowding [5]. When the capacity in one place is exceeded, the quality of life of residents and tourist experiences inevitably decline [5]. From the perspectives of inhabitants, perceived overcrowdedness can be implied from the comparison between the number of visitors of non-touristy spots and popular ones. Moreover, the extent of perceived crowding negatively influences destination attractiveness [6]. Because of the negative effects resulting from overtourism (e.g. a decrease in tourists’ loyalty [7], revisit intention [8], and satisfaction [9]), effective managing strategies are necessary for destination marketing organizations (DMOs). Recently, there has been a growing interest among scholars and practitioners in discussing the impact of overtourism [1, 10]. However, since existing studies have only examined overtourism in general pertaining to a city or a country, Koens et al. [2] underpinned the importance in relating the issue to specific attractions, to avoid overgeneralization of the results. Furthermore, earlier literature mainly analyzed overtourism via surveys/interviews with limited sample size [5]. Thus, a holistic understanding of tourist experiences when visiting overcrowded attractions merits attention. While it appears that overtourism may not exist during the global crisis, we have already witnessed domestic travel booms in few countries as a result of successful preventions of COVID-19 outbreak [11, 12]. Even during the COVID-19 pandemic, people still have desires for travelling, yet at different spots that are considered safer [13] and might be less well-known before.

To understand tourist experiences from an inductive approach, uncovering their perceptions based on user-generated content (UGC) can be beneficial. Built on the notion of experience economy, staging memorable and extraordinary experiences are crucial to the tourism industry [14]. Technology has enabled tourists to share their travel experiences in real-time [15]. The valence of online reviews can further influence consumer perceptions towards tourism products [16]. Due to the unstructured nature of online data, there is an increasing popularity in adopting topic modeling and sentiment analysis [17]. The former identifies the main topics of the reviews and is particularly suitable for exploratory studies [18]. The latter quantifies subjective information by natural language processing and computational linguistics [19].

Although text analytics of online reviews is an ideal tool to examine tourist experiences at certain attractions, insights from social media is rather scarce [20]. A comprehensive understanding towards overtourism has also been overlooked by scholars [10]. Thus, this study aims to uncover the most common issues related to overtourism when visiting overcrowded attractions and to reveal the feelings of tourists through text analytic techniques. Theoretically, this study extends the current knowledge towards overtourism by examining tourism experiences on social media. Methodologically, the technique applied in this study is beneficial to marketers who want to examine tourists’ feelings based on UGC elsewhere. Practically, destination marketers will be able to improve their management strategies accordingly, especially for emerging touristic spots during/after the COVID-19 pandemic.

## Literature Review

### Overtourism and Tourist Experiences

Overtourism occurs when hosts, tourists, or locals feel that there are too many tourists in a particular area, which leads to a decline in the quality of life of residents and worsens tourist experiences [21]. Understanding tourist experiences has long been a critical issue as tourists continue seeking for personalized, unique, and extraordinary experiences [14]. Essentially, tourists experiences refer to the interplay between tourists, destinations, and the surrounding atmosphere [22]. Although overtourism has been foregrounded in the media [10], there is an ongoing debate on the definition, antecedents, and consequences. Koens et al. [2] argue that overtourism has not been clearly defined despite its popularity. Wall [1] claims that overtourism is simply a notion based on carrying capacity, rather than a new phenomenon. Because every destination has a maximum carrying capacity, capacity standards reflect the local image and land availability [1]. Neuts and Nijkamp [23] developed a crowding perception model by examining the destination environment in Bruges. Liu and Ma [24] discovered that the perception of crowdedness is not directly related to tourist satisfaction, while Kılıçarslan and Caber [25] reported contradicting results. Notably, since the nature of service offerings and atmosphere vary among different sites, Koens et al. [2] made recommendations to analyze the effects of overtourism based on specific attractions rather than the whole destination.

Significant ramifications associated with overtourism include the deregulation of the airline industry [26], home-sharing platforms [2], and social networks that can influence tourists’ travel behavior [20]. Problems such as the increasing number of tourists in certain attractions, tourism gentrification, commodification on tourist sites, and rent escalation, are gaining more visibility [27]. These factors have all worsened tourist experiences in overcrowded areas [28]. An example can be seen from the deputy mayor’s unpleasant attitude towards tour buses in Paris [29]. The evaluation of tourist experiences in areas with a high level of perceived crowdedness could be based on the range of provided services, expenses, and the extent of security, among others [4]. Specifically, the issue of safety and security is much more difficult to manage because of the crowds [4]. Excessive waiting time as a result of too many tourists creates frustration and anxiety [30]. The perceived service quality decreases due to insufficient staff and facilities available for tourists [30]. Also, there is a relationship between perceived value, congestion, and tourist satisfaction [31]. Yet, other studies have pointed out some of the positive viewpoints. Due to the needs of interacting with other tourists when traveling [23], the presence of others positively influences tourists’ feelings [32]. Given the increase in tourist numbers, the residents in the heritage town of Besalu in Spain still recognize the economic benefits [5]. Afterall, although tourist experiences at the destination further contribute to the economy, society and the overall environment [4], recent studies have stated that crowding perception and overtourism are still neglected areas in contemporary tourism research [24, 33].

### Text Analytics on User Generated Content

To investigate tourists’ feelings when visiting overcrowded attractions, the analysis of online reviews has been recognized as a reliable source given the rich data it provides. In the digital area, consumers are more likely to share their travel experiences online [15]. Reviews posted by other tourists become more critical in influencing one’s decision-making process [34]. One typical example is TripAdvisor which enables tourists to consult reviews on any hotel, restaurant or attractions shared by other users [35]. Due to the unstructured nature of online content, text analytics try to facilitate the meaning-making process by extracting insights from social media. Particularly, Latent Dirichlet Allocation (LDA) is one of the most common topic modelling techniques that helps to deal with collections of text data [18]. LDA views a document of text as a mixture of topics that disclose words with certain probabilities. Its main purpose is to identify the underlying topics in unstructured corpus such as customer reviews [18]. Another technique, which is often incorporated with LDA is sentiment analysis [17]. Sentiments refer to feelings based on attitudes, emotions, and opinions; it determines whether an expression is positive, negative, or neutral [19]. Social media data, especially from online review platforms, are frequently used for sentiment analysis, as their content expresses the experiences and feelings of products and services [15].

Specific to this research, topic modeling uncovers visitor experiences from a bottom-up approach. As the current understanding on overtourism is limited by existing constructs and framework [2, 5], more efforts are needed to provide a holistic view of the consequences of crowdedness. The benefits of text analytics (e.g. topic modeling and sentiment analysis) have been corroborated by several studies investigating tourism experiences. For instance, Guo et al. [18] adopted LDA technique to identify the most commonly perceived factors of hotel visitors when sharing their experience online. Liu et al. [19] reported that Chinese tourists had positive sentiments regarding themes such as natural environment, landmarks, and architecture when visiting Australia. Overall, topic modeling techniques reveal the hidden insights that are often overlooked by the destination marketers [36].

## Methodology

### Study Context

France has always been a well-known destination and has been ranked number one in terms of visitor numbers over the past years [37]. Being the symbol of France, several studies proved that Paris encounters problems of overtourism [38, 39]. This study thus scrutinized the top ten cultural and heritage attractions in Paris [40]: Notre-Dame de Paris, Basilica of the Sacred Heart of Paris, Louvre Museum, Tour Eiffel, Centre Pompidou, Musée d’Orsay, City of Science and Industry, Museum of Natural History, Arc de Triomphe, and Sainte-Chapelle, and analyzed visitor experiences when travelling to attractions with an intensely high concentration of tourists.

### Data Collection and Data Treatment

To reveal issues related to overtourism when visitors travel to the above attractions, this study analyzes tourist experiences based on TripAdvisor online reviews. First, all available posts in English, of the top 10 cultural-related attractions in Paris, in TripAdvisor were extracted, resulting in a total of 140,712 posts published by any user as of the end of 2019. The following procedures were conducted in Orange 3, an open source visual programming software. Prior to the implementation of LDA topic modeling, online posts were pre-processed. A list of stopwords was prepared to eliminate non-informative text. The remaining corpus was transferred to lowercase, where diacritics were transformed to basic format. Next, text data was tokenized. All words were converted into their basic form, using lemmatization (e.g. travelling to travel).

Due to the restriction of Orange 3, only a maximum of 5,000 data instances were allowed for conducting topic modeling. Hence, 5,000 posts were randomly selected for each attraction using a random selection in excel. The sample size is similar to recent studies applying topic modeling technique [41, 42]. To ensure an equal distribution of the data, two attractions (i.e., Museum of Natural History and City of Science and Industry) having less than 300 reviews were excluded. LDA topic modelling was conducted to generate term clusters from the extracted reviews, which yielded 10 topics for each attraction based on the default setting of Orange 3. The degree of how a token contributes to a given review was revealed based on TF-IDF representation (term frequency-inverse document frequency). In the next step, based on the identified topics, a lexicon-based sentiment analysis using the Vader algorithm was adopted to extract online users’ feelings based on the posts. The results are presented by a numerical spectrum where −1 is the most negative, +1 is the most positive, and 0 suggests the neutral point.

## Results

Table 1 provides detailed descriptions of the 24 identified topics generated from LDA of the remaining eight sites. The naming of the topic was based on the top keywords with the highest TF-IDF scores detected by LDA (Table 1). This research took references from the destination image measurement [43] and overtourism risk assessment [4] to for the topic names. Though, additional topics/themes were added when the researchers encountered other important aspects during the analysis.

Unsurprisingly, visitors tend to post their experiences related to artwork, architecture, or cultural and historical background of the attraction. Nevertheless, other topics highly relevant to the effects of overtourism include, “safety and security”, “queues of customers”, “time to visit”, “social interaction”, “staff and service”, “service and facility”, “time value of money”, “visitor expectation”, “fee and ticket”, “visitor recommendations”, “emotional experience”, “reputation”, and “overall atmosphere”. Based on the keywords in Table 1, tourists often relate incidents of theft at the crowded attractions. Because of the crowds, other emerging problems include a long waiting time, unsatisfactory experiences of the provided service, occupied spaces in shops and restaurants, and insufficient toilet facilities. Consequently, when the standards did not meet visitor expectation, the disappointment from the tourists played a role in shaping their overall experiences and influencing their perceived reputation of the attractions. Furthermore, overcrowding also leads to a discussion about ticket pricing and the best time to visit. The discussion would revolve around early-bird tickets, advance booking, city pass, and free admissions during specific times or dates. Eventually, the overall tourist experiences at the sites causes visitors to reflect on the costperformance ratio in terms of time and money spent.

Among the topics relevant to overtourism, Table 2 shows that visitors felt most negatively about “safety and security”, “service and staff”, and “queues of customers”. Yet, the sentiment scores were higher regarding “social interaction”, “reputation”, and “overall atmosphere”. The results explain that although overcrowding issues might lead to frustrating touchpoints in a tourist’s journey, the experience derived from the attraction itself (e.g. appreciation towards masterpieces) can counterbalance the overall tourist experiences (e.g. high sentiment scores of “reputation” and “overall atmosphere”). Moreover, the findings demonstrate that crowds positively affect tourists’ emotional experiences and encourage interactions with others. Overall, visitors’ feelings were generally positive with a sentiment score of 0.61. However, only Notre-Dame de Paris, Basilica of the Sacred Heart of Paris, and Musée d’Orsay had an overall sentiment score higher than the average value.

**Table 1.** Summary of topics

Identified topic	Definition	Keywords
*Safety and security	Visitors’ concern about safety and security when visiting the attraction (e.g. pickpockets, security check)	dangerous, suspicious, harass, scam, pickpocket, strangers, steal, group, busy, searched
*Queues of customers	Visitors’ impression about queuing to access the attraction (e.g. length, time)	wait, inform, queue, entrance, rough, long, fast, crowd, skip, stand
*Time to visit	Visitors’ impression of what is the best time or period to visit the attraction	hours, week, time, queue, days, night time, schedule, early, summer, weather
*Social interaction	Visitors’ impression of the interaction with other tourists who are also visiting the attraction	many, groups, tourists, talk, gathering play, security, many, children, families
*Staff and service	Visitors’ impression of friendliness and helpfulness of the staff working at the attraction	staff, friendly, rude, helpful, English, nice, employees, confused, misunderstanding, refund
*Location and surroundings	Visitor’s impression about the attraction’s location and its surroundings (e.g. cleanliness, beauty)	places, landmark, Paris, city, location, distance, roads, community, cafes, area
*Service and facility	Visitors’ impression about the extra services and facilities available at the attraction	shop, restaurant, souvenir, expensive, staff, buy, pay, access, restroom, echo
*Time value of money	Visitors’ impression of the value they get in return for the money spent at the attraction	worthwhile, waiting, queue, crowds, wonderful, rewarding, special, affordable, reserve, value
*Visitor expectation	Visitors’ impression of expectation fulfilment or disappointment when visiting the attraction	disappointing, crowded, small, few, overrated, wait, queue, bucket-list, lifetime, best
*Fee and ticket	Visitors’ impression of the entry fees and tickets (e.g. prices, discounts)	price, free, ticket, enter, age, advance, early, group, cheap, pass
*Visitor recommendations	Visitors’ recommendations to other potential visitors of the attraction	incredible, recommend, visit, return, must, advise, tip, overrated, impression, worth
*Emotional experience	Visitors’ emotions experienced throughout their visit to the attraction	disappointing, amazing, remarkable, unique, worth, awesome, breathtaking, boring, surprising, fun,
*Reputation	Visitors’ impression of the fame and reputation of the attraction and its impact on the visitor experience	famous, overrated, rewarding, unique, worth, overcrowded, icon, expect, nice, better
*Overall atmosphere	Visitors’ impression of the atmosphere in and at the attraction (e.g. cozy, relaxing)	neighborhood, atmosphere, cozy, worth, views, outside, crowd, romantic, sparkles, light
Navigation design	Visitors’ ability to navigate and walk through the attraction	signs, lost, English, navigate, understand, helpful, way, display, walking, confusing
Scenery and view	Visitors’ impression about the scenery and views as seen from and around the attraction	view, good, amazing, scenery, top, city, surrounding, spiritual, steep, climb
Accessibility	Visitors’ impression about the general accessibility of the attraction (e.g. wheelchair, elderly people)	climb, metro, steps, stairs, lift, tired, walk, base, easier, passageway
Exhibition impression	Visitors’ impression of the exhibitions they visited at the attraction (e.g. design, appeal)	collection, impressionism. Monet, sculptures, exhibition, interesting, artists, amount, huge, section
Architecture impression	Visitors’ impression of the architecture of the attractions (e.g. building, glasswork)	chapels, scope, building, inside, style, ceiling, treasure, design, pyramid, shape
Artwork impression	Visitors’ impression of the artwork present at the attraction (e.g. interesting, inspiring)	interesting, collection, art, Monet, painting, gallery, design, glasswork, sculptures, Picasso
Information and education	Visitors’ impression of the information and educational value provided at the attraction	information, signs, children, read, understand, school, story, boards, languages, cards
Culture and history	Visitors’ impression and awareness of the cultural and historical background of the attraction	story, shape, magnificent, cultural, memorial, artefacts, Napoleon, old, history, past
Impact of religion	Visitors’ impression of the impact of their own religion on the visitor experience at the attraction	church, religious, significance, priest, service, pray, Christian, believer, worship, inspiring
Public transport	Visitors’ impression of the public transport available to arrive at the attraction	public, metro, booth, transit, bus, arrive, easy, transport, access, distance

Note: *topic is related to overtourism

**Table 2.** Summary of sentiment scores.

	NDP	BSH	LM	TE	CP	MO	AT	SC	Count	Average
*Safety and security	–	0.00	–	0.61	–	–	–	–	2	0.31
*Queues of customers	0.61	–	0.51	0.48	–	–	–	0.45	4	0.51
*Time to visit	–	0.65	0.53	0.59	–	0.77	–	–	4	0.64
*Social interaction	0.69	0.82	–	–	0.67	–	–	–	3	0.73
*Staff and service	–	–	–	–	0.81	–	−0.14	–	2	0.34
*Location and surroundings	0.67	0.57	–	0	0.75	–	0.66	–	5	0.53
*Service and facility	–	–	–	–	0.68	0.73	0.51	0.29	4	0.55
*Time value of money	–	–	–	0.48	0.72	0.73	–	0.35	4	0.57
*Visitor expectation	–	–	0.71	0.46	–	–	–	–	2	0.59
*Fee and ticket	–	–	0.70	0.51	–	0.67	0.67	0.55	5	0.62
*Visitor recommendations	0.76	–	–	0.69	–	0.64	–	0.45	4	0.64
*Emotional experience	–	–	0.64	0.84	0.74	0.63	–	–	4	0.71
*Reputation	0.80	–	–	–	–	–	–	–	1	0.80
*Overall atmosphere	–	0.79	–	0.84	–	–	–	–	2	0.82
Navigation design	–	–	0.39	–	0.23	0.70	–	–	3	0.44
Scenery and view	–	0.68	–	–	0	–	0.74	0.56	4	0.50
Accessibility	0.70	0.61	–	–	–	–	0.37	–	3	0.56
Exhibition impression	–	–	0.51	–	–	0.7	–	–	2	0.61
Architecture impression	0.68	0.66	0.37	–	0.69	–	0.83	0.56	6	0.63
Artwork impression	0.67	–	0.74	–	0.78	0.69	0.40	0.55	6	0.64
Information and education	–	–	–	–	–	–	0.56	0.76	2	0.66
Culture and history	0.78	–	0.72	–	–	0.79	0.44	0.72	5	0.69
Impact of religion	0.77	0.85	–	–	–	–	–	–	2	0.81
Public transport	–	0.87	–	–	–	–	–	–	1	0.87
Average	0.71	0.65	0.58	0.55	0.61	0.71	0.50	0.52		0.61

Note: NDP = Notre-Dame de Paris, BSH = Basilica of the Sacred Heart of Paris, LM = Louvre Museum, TE = Tour Eiffel, CP = Centre Pompidou, MO = Musée d’Orsay, AT = Arc de Triomphe, SC = Sainte-Chapelle; topics sorted based on average sentiment scores (from small to large); count: the number of attractions accorded a score; *topic is related to overtourism

## Conclusion

### General Discussion

As the demand for leisure and the plethora of tourist destinations has increased, especially emerging at the domestic level, overtourism has become a global issue. Although literature has highlighted the multifaceted problems resulted from overtourism such as safety and security [33], poor management strategies [2], and negative tourist experiences [30], crowding perception and the concept of overtourism have not been adequately addressed to date [10, 24]. Additionally, a strong focus was put on investigating the perception of residents [4, 44] rather than providing a holistic understanding towards tourist experiences related to overtourism. Since overcrowdedness occurs in site-specific terms [2], this study is a much more detailed one as it scrutinizes the most popular attractions perceived as overcrowded.

First, the lowest sentiment score of safety issue might imply tourist’ uncertainty and anxiety [30] owing to the high number of tourists. This result can be explained via several negative words (e.g. “dangerous”, “scared”, and “nervous”) when tourists described their experiences at the sites. Indeed, it is unsurprising that tourists are concerned about their safety when traveling. This point has also been brought up in recent literature [33]. Buhalis [45] suggests that visitor safety should be included in the comprehensive strategies developed by the DMOs to have a prosperous and sustainable destination. Other salient issues resulting from overtourism include the poor performance of service, staff, and insufficient facilities. Consistent with earlier studies investigating the effect of crowdedness on service performance and consumer perceptions [46], this research discovered that visitors potentially perceived service quality as compromised due to the presence of crowds, leading to an unsatisfactory outcome. Moreover, this study highlights the excessive waiting time at the attractions that can cause negative tourist experiences. Apart from implementing possible solutions to reduce the perceived waiting time such as embedding gamifying and technological elements to entertain visitors [47], based on the keywords derived from this study, it appears that tourist experiences can be improved by informing one in advance of a rough waiting time or providing sufficient seating capacity. Notably, it is interesting to see that a high level of perceived crowdedness is not always negative [46]. Specifically, a feeling of co-presence with other tourists [48] fosters the chances of interacting with one another during the trip. This is conveyed by the higher sentiment score of “social interaction” and “overall atmosphere”. Suggested by Neuts and Nijkamp [23], the perceived crowding level is related to the interactions with other tourists, which subsequently influence tourists’ feelings [32]. While it appears that tourists’ feelings are generally positive when visiting the overcrowding attractions, the high sentiment scores were mainly contributed by topics that may not have a direct relationship with the crowds (e.g. “artwork impression”, “culture and history”). This assumption is especially obvious at the Arc de Triomphe where visitors were most positive towards the architecture while being negative towards the service and staff. Similarly, queuing received the lowest sentiment score whereas the reputation and the history of the attraction received the highest sentiment score at Notre-Dame de Paris. Echoing the study of Capocchi et al. [33], although the dimensions related to overtourism are interrelated and overlapping, it is evident that overtourism is a problem that must be addressed to improve the quality of tourist experiences and to ensure the sustainability of attraction development. Despite that the number of international tourists in popular attractions declined during the global crisis, domestic tourism is booming [12]. Indeed, while it might take years until the tourism industry recovers from the COVID-19 pandemic, people are still traveling in the meantime yet in different forms (e.g. road trips, short-distance travel). Well-known destinations might be less attracted to tourists, but the phenomenon of overtourism is likely to occur in other spots that were less promoted and unpopular previously.

### Theoretical Contributions and Practical Implications

Given that a high number of visitor arrivals appears to be profitable, tourist experiences end up being compromised and negative feelings at specific attractions emerge concurrently. This research provides a threefold contribution to theory, methodology, and practice. First, this study is novel in that it links the emerging fields of overtourism and social media [20] into tourist experiences. Unlike earlier research built upon existing measurements/figures [4, 44], this study takes one step further by exploratorily discovering the critical dimensions in managing tourist experiences at overcrowded attractions. Seeing that earlier literature often examined perceptions of crowding based on tourists’ demography (e.g. age and gender) [32], this study sheds light on the potential directions for future research to test the identified topics based on visitors’ socio-demographic backgrounds. Additionally, this study also contributes from a methodological angle by incorporating topic modelling technique and sentiment analysis to reveal tourists’ subjective perceptions. Practically, the findings provide insights to attraction management, DMOs, governments, and relevant stakeholders to strategically plan and enhance both the tourist experiences and socio-ecosystem. Because of the international travel restrictions, overtourism might not be highly relevant in the post-COVID-19. However, it is critical to be aware of the localized overcrowding as a new occurrence in the tourism industry. This study speaks to the thriving global tourism industry in that it offers a detailed account regarding tourist experiences and their feelings when traveling to overcrowded attractions.

### Limitations and Recommendations for Future Research

As with any research, this study is not without limitations. First, this research only investigates visitors’ experiences in the top attractions in Paris, leading to a high degree of uncertainty in the generalization of results. Future studies should be done that examine other crowded attractions. Meanwhile, comparing the results with locations that are less crowded could further reveal more insights. Moreover, the findings in this research are based on online reviews from TripAdvisor. Future studies should consider other platforms such as Facebook and Instagram. Additionally, this research did not consider reviews posted by non-English speakers, while they could be emerging markets (e.g. Chinese) for the tourism industry. Methodologically, some attractions may have numerically fewer or more topics rather than ten. It therefore leads to an unequal distribution regarding the number of posts in each identified topic. Finally, researchers are suggested to employ various topic modeling techniques (e.g. correlation explanation and k-means) to find the optimized results. Apart from understanding the valence of online reviews, future studies can also incorporate emotional analysis to better quantify tourists’ emotional engagement in the context of overtourism.
